# Viral Metagenomic Analysis of Bat Fly Pupae

**DOI:** 10.3390/pathogens15090950

**Published:** 2026-09-07

**Authors:** Levente Láng, Gábor Kemenesi, János Novák, Zsófia Lanszki, Tamás Görföl

**Affiliations:** 1 National Laboratory of Virology, Szentágothai Research Centre, University of Pécs, 7624 Pécs, Hungary; lang.levente@edu.pte.hu (L.L.); kemenesi.gabor@pte.hu (G.K.); novakjanos01@gmail.com (J.N.); lanszki.zsofia@pte.hu (Z.L.); 2 Institute of Biology, Faculty of Sciences, University of Pécs, 7624 Pécs, Hungary; 3 Department of Systematic Zoology and Ecology, Eötvös Loránd University, 1117 Budapest, Hungary

**Keywords:** bat flies, virome, metagenomics, vertical transmission, vector competence, zoonotic viruses

## Abstract

Bats are important reservoirs of zoonotic viruses, yet the role of their obligate ectoparasites, particularly bat flies, in viral maintenance and transmission remains poorly understood. Although viral sequences have been detected in adult bat flies, vertical transmission to their offspring has not been investigated. Here, we present the first metagenomic analysis of bat fly pupae to assess transgenerational viral persistence. Eighty pupae associated with a large *Miniopterus schreibersii* colony in Hungary were analyzed by next-generation sequencing. We generated more than 69 million reads and over 28,000 assembled contigs. Although initial classification suggested the presence of viral sequences, none were confirmed following sequence-level validation. These findings highlight the substantial potential for false-positive viral assignments in metagenomic datasets and the importance of rigorous post-classification validation. While our results provide no evidence that vertical transmission is a major mechanism of viral maintenance in bat–bat fly systems, this conclusion is limited by the relatively small sample size and single sampling occasion. Highly divergent or low-abundance viruses may also have remained undetected. Broader sampling across seasons and locations is therefore needed to assess the generality of these findings. Nevertheless, this study provides a valuable first step toward understanding potential transgenerational viral persistence in bat flies and highlights the need for complementary approaches to clarify their role in viral transmission.

## 1. Introduction

Bats are the second largest order among mammals, exhibiting remarkable diversity worldwide. They are progressively associated with a wide range of pathogens such as SARS-CoV-2, Marburg virus, and Nipah virus [1]. Furthermore, several studies support the premise of bats being potential reservoirs of the highly pathogenic Ebola virus [2,3], which has caused multiple outbreaks in tropical Africa in the past decades. Besides the various viral pathogens, they host several types of ectoparasites, including bat flies (Diptera: Hippoboscoidea), bedbugs (Cimicidae), ticks (Argasidae and Ixodidae), fleas (Ischnopsyllidae), and mites (Acari) [4].

Bat flies are the most abundant ectoparasites, categorized into two families, namely Nycteribiidae and Streblidae, with the former exhibiting higher diversity in the Eastern Hemisphere, while Streblidae are more abundant in the Western Hemisphere [5]. Both families are members of the Hippoboscoidea superfamily and harbor 275 and 227 species, respectively. According to our current knowledge, only 16 species of Nycteribiidae and one Streblidae species are reported from Europe [6]. These obligate blood-feeding arthropods show a close ecological association with bats, living in their fur and under their wing membrane.

Relatively little is known about the life history of bat flies; generalizations are based on limited studies of a few species (i.e., *Basilia hispida*). Bat flies exhibit a rather specialized mode of reproduction uncommon among insects. Following internal fertilization, a single larva develops through all larval stages inside the female, sustained by secretions from maternal milk glands. Females deposit the third-instar larvae on the roost walls, where the larvae immediately form a puparium, and after 3–4 weeks, the adult fly emerges seeking a host. Cases have been reported of larvae deposited on the host’s body; however, the pupae either failed to develop or were groomed off by the bat. Adult flies must locate a host within 1–3 days, as they cannot survive unfed beyond this period. The total lifespan of *B. hispida* differs between sexes; males live around 136 days, while females live 195 days, including pre-partum and larval stages [7].

Long-term co-evolution with their hosts resulted in special morphological and behavioral adaptations, such as winglessness (Nycteribiidae) and eyelessness, giving them a “spider-like” appearance [5]. Another consequence of the co-evolution is the high host specificity of bat flies [8], which is a key consideration in studies focusing on the vectorial potential of parasites. Bat flies also exhibit a remarkable host fidelity and typically remain on the same bat throughout their entire lifespan. Nevertheless, in communal roosts, host switching can occur as a result of events such as returning from deposition of the larva on the roost wall, potentially facilitating the spillover of a pathogen among bat individuals intra- or inter-specifically, promoting the spread of the pathogen [5]. Moreover, vertical transmission may also occur if the reproductive organs or maternal milk glands are infected, facilitating the transmission of the virus into the developing larvae and subsequently the next generation.

The pupal stage is therefore of particular relevance for investigating vertical transmission in bat flies. Unlike adult bat flies, which may acquire viral material through feeding on an infected host or through other horizontal routes, pupae represent a developmental stage produced by the previous generation and deposited on the roost substrate. Consequently, detection of viral sequences in pupae could provide evidence consistent with the persistence of viruses from the reproductive female into her offspring, rather than merely reflecting recent acquisition from an infected bat host. Investigating pupae therefore provides a complementary approach to previous studies based primarily on adult bat flies, in which the presence of viral sequences alone cannot readily distinguish between horizontally acquired viral material and transgenerational persistence. Furthermore, as pupae remain attached to the roost substrate during development, they can be sampled independently of their adult hosts, providing an opportunity to investigate viral persistence across the developmental transition from one generation to the next.

Previous studies documented the presence of a diverse array of bacterial, fungal, and viral pathogens in bat flies, reinforcing the hypothesis that these insects possess vectorial properties. The efficiency of bat flies as vectors is dependent on whether they act as mechanical transporters or stable biological reservoirs for the pathogens. Although bat flies are highly specialized ectoparasites and are known to be only associated with bats, they can share a host with other types of parasites, such as Ixodid bat ticks, which are capable of also feeding on humans [9], thereby increasing the potential of spillover.

The bat–bat fly–virus hyperparasitic system remains highly understudied, and the vectorial capacity of bat flies has yet to be clarified, although it is a very important factor in viral ecology. To the best of our current knowledge, no study has investigated the virome of bat fly pupae before; consequently, an exploratory investigation regarding this context could provide valuable insights into host–virus interactions, potentially revealing important information about the ectoparasite’s vectorial potential. To address this gap, next-generation metagenomic sequencing serves as a robust molecular biological tool for viral surveillance by providing an agnostic platform for pathogen discovery. In contrast to targeted diagnostics, metagenomics enables concurrent detection of viruses within one sample, offering high-resolution surveillance. Peerless execution requires rigorous sample preparation, accurate high-throughput sequencing, and proper bioinformatic processing aided by correct databases.

## 2. Materials and Methods

### 2.1. Sample Collection

**Figure 1:**
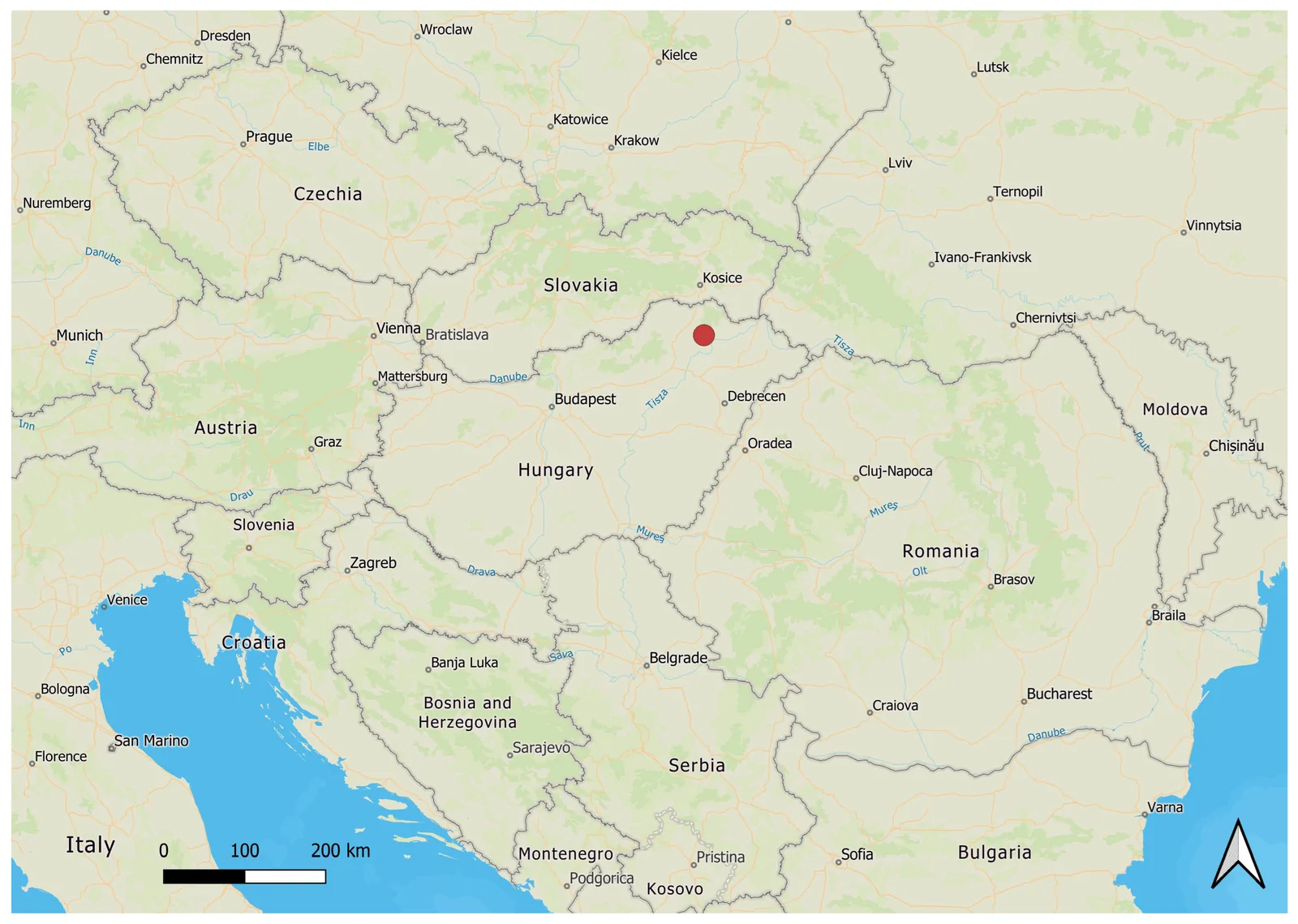
Map showing the sampling locality (red dot) in northeastern Hungary.

**Figure 2:**
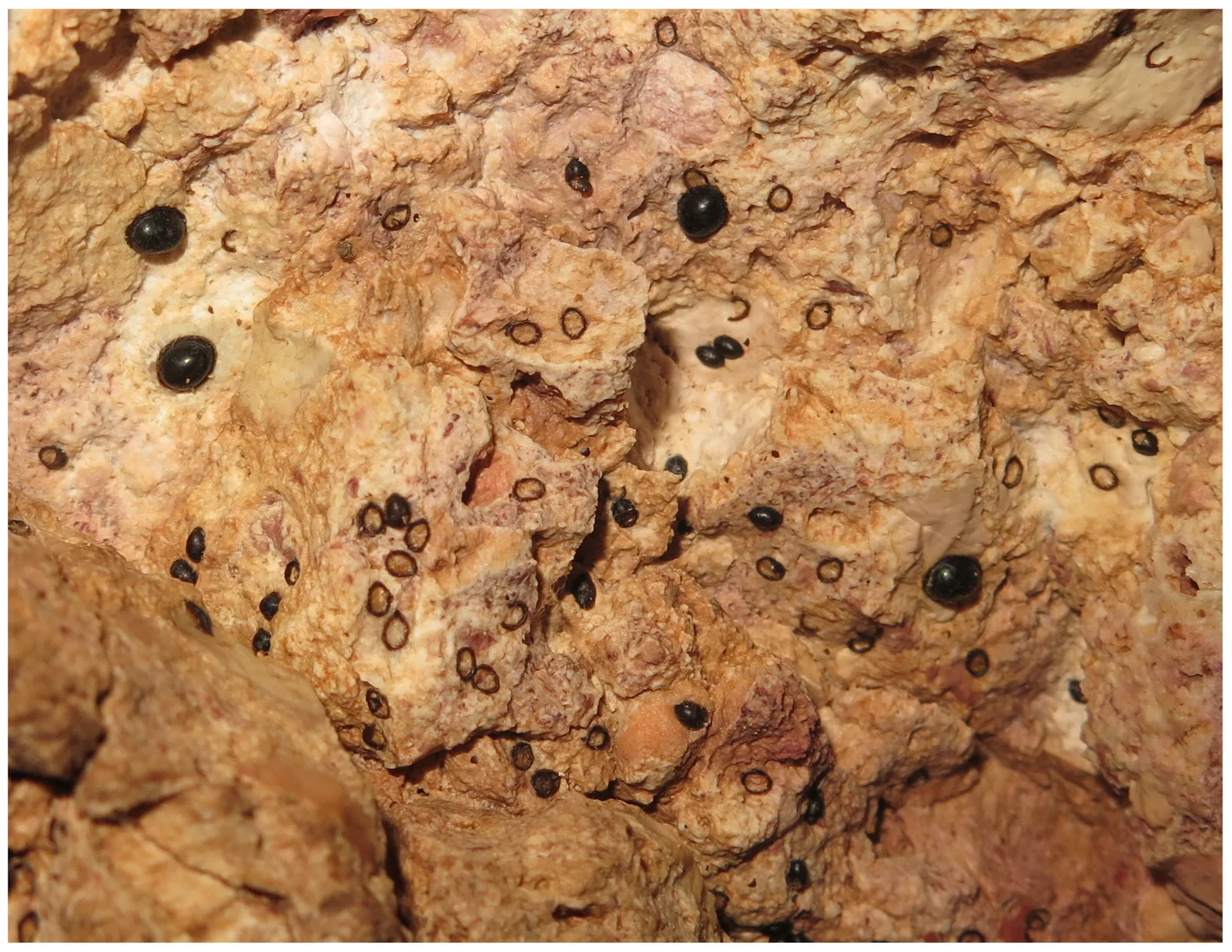
Bat fly pupae of different sizes on the mine ceiling. Note the characteristic remains of already hatched pupae on the wall.

The site of sample collection was an abandoned mine, which hosts one of the largest bent-winged bat (*Miniopterus schreibersii*) colonies in Hungary (Figure 1). While *M. schreibersii* is the predominant bat species, the mine is a multi-species roost also frequently used by *Myotis blythii*, *M. myotis*, and *Rhinolophus ferrumequinum*. The local *M. schreibersii* bats are hosts of three bat fly species: *Nycteribia schmidlii*, *Penicillidia dufourii*, and *P. conspicua*. Bat fly pupae were collected from the roost walls where usually only *M. schreibersii* bats are roosting and grouped by size, as morphological identification is not possible in the pupal stage (Figure 2). The size of the bat fly pupae correlates with the size of mature bat flies; small pupae likely represent *N. schmidlii*, and large pupae likely represent *P. dufourii* and *P. conspicua*. A total of 8 pools (4 small, 4 large) were established, each pool containing 10 pupae to provide sufficient biomass for viral analysis. Samples were transferred to the laboratory in a −18 °C portable freezer and put into a −80 °C freezer for three months until processing. A previous study in our laboratory has proven the presence of Lloviu virus (LLOV) at the mine in several samples (blood, feces, etc.) and ectoparasites (bat flies and ticks) of *M. schreibersii*, making this mine the perfect candidate for our research [10].

### 2.2. Viral Nucleic Acid Extraction

The pupae were washed with PBS to remove pollutants and dust originating from the cave wall. To ensure the most efficient homogenization, 50 μL PBS and three glass beads were added to each sample. Homogenization was performed for 10 min at maximum frequency (30/s) with a Qiagen TissueLyser II (Qiagen, Hilden, Germany). Subsequently, the samples were diluted with an additional 150 mL of PBS, vortexed, and centrifuged. A 160 μL aliquot of the supernatant of each sample was subjected to viral enrichment according to the following protocol [11]: supernatants were filtered through 0.8 μm pore size filters, and the flow-through was transferred into new Eppendorf tubes. Thereafter, a DNase treatment cocktail was added, containing 15.5 μL of TURBO DNase buffer, 4.5 μL of TURBO DNase (Thermo Fisher Scientific, Waltham, MA, USA), 2 μL of Bensonase Nuclease (Sigma-Aldrich, Burlington, MA, USA), and 1 μL of Micrococcal Nuclease (Thermo Fisher Scientific, Waltham, MA, USA), and the mixture was incubated at 37 °C for 2 h. Total viral RNA was isolated using the QIAamp Viral RNA Mini Kit (Qiagen, Hilden, Germany) following the manufacturer’s instructions, with specific modifications. Following the incubation period, the entire volume of each sample was used for extraction. To ensure compatibility with sequencing, the RNA was eluted in 30 μL of nuclease-free water rather than the provided elution buffer. A negative control was included to exclude potential laboratory contamination, and several other types of samples were successfully sequenced alongside the bat pupae.

### 2.3. cDNA Synthesis, Random Amplification, and Sequencing

The cDNA synthesis was performed by employing a previously published protocol [12]. K8N random primers were used; 1 μL of primer (2 μM) and 1 μL of dNTP were added to 11 μL of the samples and incubated for 5 min at 65 °C. To this mix, 4 μL of SuperScript IV buffer, 1 μL of SuperScript IV reverse transcriptase enzyme (Thermo Fisher Scientific, Waltham, MA, USA), 1 μL of dithiothreitol (DTT), and 1 μL of RNase inhibitor (Thermo Fisher Scientific, Waltham, MA, USA) were added per sample, and the reaction was run in the thermocycler (Thermo Fisher Scientific, Waltham, MA, USA) to produce cDNA, using the following protocol: 4 min at 25 °C, 50 min at 42 °C, 10 min at 70 °C, and eventually cooling to 4 °C. To the product, 1 μL of K8N primer (10 μM), 1 μL of dNTP, and 3 μL of Klenow Fragment buffer (New England Biolabs, Ipswich, MA, USA) were added and incubated at 95 °C for 3 min. Finally, 1 μL of Klenow Fragment (New England Biolabs, Ipswich, MA, USA) was added to the product of the previous mixture and incubated at 37 °C for 60 min, followed by 10 min at 75 °C, and then cooled to 4 °C.

Amplification of the double-stranded DNA was carried out by mixing 1 μL of dNTP, 1 μL of K8 random primer, and 0.25 μL of Q5 High-Fidelity DNA polymerase with 5 μL of 5x Q5 High-Fidelity Mastermix (New England Biolabs, Ipswich, MA, USA), diluted to a final volume of 20 μL with nuclease-free water. Then, 5 μL of template was used from the previous reaction for amplification and denatured at 98 °C for 3 min, followed by 35 cycles of 98 °C for 30 s, 55 °C for 30 s, and 72 °C for 5 min, with a final elongation of 5 min at 72 °C before cooling to 4 °C. The amplification product was cleaned with AMPure XP magnetic beads (Beckman Coulter, Brea, CA, USA) following the manufacturer’s protocol. Sequencing was performed on the Illumina NovaSeq platform by Novogene Ltd. (Cambridge, UK), and adapter-trimmed paired-end reads (2×150 bp) were used for subsequent analyses.

### 2.4. Bioinformatic Analysis

Following data acquisition, quality control and adapter trimming were performed on the raw reads using FastQC v0.12.0 (https://www.bioinformatics.babraham.ac.uk/projects/fastqc/ accessed on 24 June 2026) and fastp v1.01 software [13]. SISPA adapters were removed with Porechop v0.2.4 (https://github.com/rrwick/porechop accessed on 24 June 2026). The filtered, high-quality reads for each sample underwent de novo assembly using MEGAHIT v1.2.9 [14], filtering for a minimum contig length of 500 bases. CheckV v1.0.3 [15] was applied to check the quality and completeness of contigs of possible viral origin. For taxonomic binning, Kaiju v.1.10.1 [16], Kraken2 v2.1.2 [17], and Diamond v0.9.24+MEGAN v6.25.9 [18,19] were applied.

All reads and contigs assigned to viral taxa by the metagenomic classification tools were subjected to manual validation to distinguish potentially genuine viral sequences from false-positive assignments and sequences originating from contamination or other non-viral sources. Candidate sequences were individually examined and searched against the NCBI non-redundant nucleotide (nt) database using BLAST (https://blast.ncbi.nlm.nih.gov/Blast.cgi accessed on 24 June 2026). The resulting hits were manually evaluated based on taxonomic identity, sequence similarity, and alignment coverage. In a number of cases, BLAST searches indicated that sequences initially classified as viral were more likely to originate from bacterial, host genomic, or other non-viral sequences. Such sequences were excluded from further analysis.

Candidate sequences and contigs that remained in the potentially viral category after BLAST-based assessment were further evaluated by mapping the quality-trimmed and adapter-removed reads and assembled contigs against the sequences from BLAST results using Minimap2 and Bowtie2, respectively. Sequences showing positive mapping were subsequently manually inspected in Geneious Prime 2026.1.1 to exclude potential spurious matches. This step was used to determine whether the putative viral signal was consistently supported by the underlying sequencing reads and whether the observed pattern was compatible with a genuine viral sequence rather than a sequencing artifact, database-related misclassification, or other bioinformatic artifact.

## 3. Results

A total of 8 pools (n = 80) of bat fly pupae were processed and analyzed. Next-generation sequencing generated 69,207,932 reads (average number per sample: 8,650,991.5) that were assembled into 28,669 contigs of 500+ bp in length (average number per sample: 3583.6 and average length: 862.75 bp) (Table 1).

Notably, the metagenomic analysis yielded several positive hits with various metagenomic programs, but after careful manual investigation, these proved to be false-positive results; hence, we could not find any true viral contigs in the sample pools.

## 4. Discussion

Given that viruses represent the most abundant and diverse group of zoonotic pathogens, this study focused on evaluating the viral vectorial capacity of bat flies. Although bat flies exhibit a high degree of host specificity, their close ecological association with mammalian hosts makes them significant yet understudied contributors to the maintenance and transmission of viral lineages. Metagenomic investigations have identified several novel viral species within bat flies, including an orthoreovirus (family *Reoviridae*) isolated from *Eucampsipoda africana* in South Africa [20], which was the first orthoreovirus detected in arthropods, whose natural hosts are typically vertebrates. The same Nycteribiid bat fly species was also found to harbor a novel virus from the *Peribunyaviridae* family, which was genomically characterized as being most closely related to two bat viruses [21]. Additionally, a novel ledantevirus (family *Rhabdoviridae*), Kanyawara virus, was identified in Ugandan Nycteribiid bat flies [22]. These results, along with the previous successful detection of the Lloviu virus in bat flies [10], raise speculation regarding their potential role as vectors.

Bat flies exhibit adenotrophic viviparity, in which a single larva develops within the female and is nourished by secretions from specialized milk glands until the third-instar larva is deposited as a non-feeding prepupa on the roost substrate, where it rapidly forms a pupa. Because the pupal stage does not feed after deposition, microorganisms detected in newly deposited pupae are unlikely to have been acquired through feeding from the external environment, making maternal or transovarial transmission a biologically plausible route for heritable microorganisms in bat flies. Indeed, vertically inherited bacterial endosymbionts have been detected in bat fly pupae, including pupae collected shortly after deposition and before substantial contact with the cave substrate; identical or closely related symbiont sequences in females and pupae provided evidence for maternal transmission [23].

Direct evidence for vertical transmission of viruses into bat fly pupae remains limited, because most virological studies have analyzed adult flies or pooled ectoparasites rather than pupae. Nevertheless, several observations support this possibility. A novel sigmavirus was detected in all 20 *Cyclopodia greefi* bat flies collected from an *Eidolon helvum* bat in Nigeria, whereas the virus was not detected in the corresponding bats. Since sigmaviruses are generally recognized as insect-associated viruses that may be transmitted vertically in dipterans, the high prevalence of this virus in bat flies is consistent with the possibility that related viruses could be maintained through bat fly development and occur in pupae; however, the study did not directly examine pupae or demonstrate maternal transmission [24]. Stronger, although still indirect, evidence comes from a study of ledanteviruses in nycteribiid bat flies in Uganda. That study detected 21 ledantevirus variants in bat flies from a single roost, and virus-host cophylogenetic analyses supported virus-fly codivergence, leading the authors to propose that vertical transmission is the predominant mode of transmission among bat flies [25]. The cophylogenetic evidence is compatible with transmission through the maternal lineage, but it does not by itself demonstrate the presence of ledantevirus RNA or infectious virus in individual pupae.

Conversely, vertical transmission is not necessarily a characteristic of all viruses detected in bat flies. In experimental studies of *Eucampsipoda africana*, first-filial-generation pupae and adult flies were consistently negative for Marburg virus RNA after exposure of flies to infected *Rousettus aegyptiacus* bats, providing no evidence for vertical transmission of Marburg virus in this system [26]. Other viruses, including Mahlapitsi virus, an orthoreovirus, and Wolkberg virus, an orthobunyavirus, have been isolated from *E. africana* associated with *R. aegyptiacus* in South Africa, but vertical transmission into pupae was not demonstrated for either virus [20,21]. Thus, the presence of a virus in adult bat flies does not necessarily indicate that it is vertically transmitted or maintained throughout metamorphosis.

Although metagenomic sequencing provides substantial opportunities for viral surveillance and discovery, the interpretation of metagenomic datasets remains challenging, particularly because viral sequences are often present at low abundance, highly diverse, and poorly represented in reference databases. A major challenge is therefore balancing sensitivity and specificity during taxonomic classification. In our analysis, this resulted in a considerable number of initial viral assignments that could not be confirmed during subsequent validation, highlighting the potential for false-positive detections in viral metagenomic studies.

Most taxonomic classification approaches rely on similarity to sequences or proteins present in reference databases. This introduces an inherent bias toward well-characterized organisms and viruses, while highly divergent or previously undescribed viruses may remain undetected or may be assigned to an inappropriate taxon [27]. Although virus-specific databases can reduce the influence of the broader database bias, they cannot fully overcome the limited representation of viral diversity. This is particularly relevant for metagenomic studies of non-model hosts and environmental samples, where the true viral diversity is likely to be substantially greater than that represented in current reference databases.

The different classification approaches used in our analysis also illustrate the general limitations of reference-based taxonomic assignment. Nucleotide- and k-mer-based methods can provide sensitive detection but may generate spurious assignments when short reads or contigs share limited sequence similarity with reference genomes. Protein-based approaches can improve the detection of more divergent viral sequences, but they remain dependent on the availability and quality of reference proteins and may be affected by ambiguous matches. Alignment-based searches can provide additional evidence, yet short or low-complexity sequences may still produce misleading hits. Thus, no individual classification method can be considered sufficient for reliable viral identification.

Our results emphasize the importance of subsequent sequence-level validation of candidate viral hits. Mapping reads and contigs against known viral sequences (NCBI) provides a more stringent assessment by considering the distribution and extent of sequence coverage rather than relying solely on individual similarity matches. In combination with manual examination of candidate contigs and their genomic context, this approach allowed us to distinguish likely true viral sequences from spurious taxonomic assignments. Such validation is particularly important when investigating novel viruses, where limited similarity to known viruses can make automated classification inherently uncertain. The need for additional validation and follow-up analyses to exclude false-positive viral detections has also been emphasized previously [28]. We therefore recommend that taxonomic classifications obtained from metagenomic datasets should be regarded as preliminary evidence and supported by independent sequence-level validation before being interpreted as evidence of viral presence [29].

As the first study to address this question, the present research aimed to characterize the virome of bat fly pupae to further investigate their vector capacity. While negative results may suggest the absence of vertical transmission of viral pathogens to subsequent generations in bat flies, this conclusion should be interpreted with caution. It remains possible that the lack of detectable viruses in pupae reflects limitations of the sampled dataset rather than true biological absence.

Our study was based on a single sampling occasion, during which 80 pupae were collected from mine walls within a relatively restricted area. Given their close spatial proximity, the pupae may have originated from the same or a limited number of bat groups, thereby providing only a narrow representation of the bat fly population. Furthermore, sampling was conducted at the end of the bats’ hibernation period and therefore represents only a single point in the seasonal dynamics of virus circulation. This may be particularly relevant in the context of Lloviu virus circulation in the studied *Miniopterus schreibersii* population. Regular bat mortality events, presumably associated with LLOV, occurred in the mine during this period of the year. In contrast, apparently healthy living bats infected with LLOV, as well as bat flies carrying the virus, were detected during August–October, despite regular sampling also being conducted in April–May and July [10,30]. This temporal variation illustrates how the timing of sampling may substantially influence the detection of viruses and emphasizes the importance of repeated sampling across different seasons. A larger sample size and sampling at multiple time points would therefore provide a more comprehensive assessment of the viral diversity present in bat fly pupae and increase the likelihood of detecting viruses occurring at low prevalence or abundance.

From a biological perspective, the absence of confirmed viral sequences in the pupae should also be considered in the context of the generally low and highly variable prevalence of many viruses in bat blood. Unlike viruses that are actively shed through the gastrointestinal tract and can consequently reach relatively high percentages in fecal material, viruses circulating systemically or present in the blood may occur only transiently and at low prevalence in apparently healthy bats. This difference can substantially influence their detectability and, consequently, their probability of being acquired by hematophagous ectoparasites. For example, in a multi-year study of *Miniopterus schreibersii* in Hungary, Lloviu virus RNA was detected in only ~1% of live-sampled bats, with positive samples restricted to particular sampling periods. The positivity rate among the ectoparasites was even slightly lower than that observed in the bats [10].

In addition, methodological limitations may hinder the detection of highly divergent viruses, particularly when they are present at very low titers. Viral enrichment can increase the relative proportion of viral nucleic acids by reducing the contribution of host and other non-viral nucleic acids, such as those of bacterial origin. However, even after enrichment, non-viral nucleic acids may constitute a substantial proportion of the sequencing material, potentially masking viruses present at very low abundance.

Taken together, these limitations prevent us from concluding with certainty that bat flies do not contribute to the vertical transmission of viruses. Definitively addressing this question would likely require controlled experiments using laboratory-maintained bat fly colonies, allowing viral transmission between successive generations to be directly monitored under controlled conditions. Nevertheless, our study provides an important contribution to understanding the potential role of bat ectoparasites in viral transmission. In particular, the use of pupae represents a novel approach for investigating whether viruses may be maintained and transmitted across generations in bat-associated arthropods. Although our findings did not provide evidence for such transmission in the investigated population, they establish a basis for future studies combining broader temporal and geographic sampling or controlled experimental approaches. Such investigations will be essential for clarifying the role of bat flies in the maintenance and transmission of viruses within bat–ectoparasite systems.

## 5. Conclusions

In summary, our metagenomic analysis did not detect any known relevant viruses in bat fly pupae, providing no evidence that vertical transmission is a major mechanism for the maintenance of viruses across generations of these arthropods. However, this finding should be interpreted cautiously, as the study was based on a relatively small number of pupae collected during a single sampling occasion and from a geographically restricted area. In addition, highly divergent or low-abundance viruses may have remained undetected despite viral enrichment and metagenomic sequencing. These limitations mean that the absence of detected viruses cannot be considered definitive evidence against vertical transmission.

Consequently, while our results suggest that vertical transmission is unlikely to be the primary mechanism maintaining viruses in bat fly populations, they do not exclude a potential role of bat flies in viral maintenance or transmission. Further studies incorporating larger sample sizes and repeated sampling across different seasons and locations would provide a more comprehensive assessment of viral presence in bat fly pupae and help determine whether the negative findings observed here are consistent across populations and time. Ultimately, controlled transmission experiments using laboratory-maintained bat fly colonies would provide the strongest evidence for or against vertical transmission, although establishing and maintaining such colonies represents a considerable technical challenge. Furthermore, the substantial number of initial false-positive taxonomic assignments observed in our analysis highlights the importance of rigorous sequence-level validation. In the absence of a universally accepted gold standard for viral metagenomic analysis, combining automated classification with careful manual validation remains essential for reliable interpretation of viral metagenomic datasets.

## Figures and Tables

**Table 1:** Sequencing and de novo assembly results of the eight pools.

Pool Code	Read #	Contigs # (500+ bp)
GT102	6,162,586	3087
GT103	5,801,944	3075
GT104	5,129,648	5274
GT105	7,611,662	6366
GT106	7,917,366	2538
GT107	9,349,996	3987
GT108	11,371,342	3275
GT109	15,863,388	1067
**Total**	**69,207,932**	**28,669**

## Data Availability

The raw data supporting the conclusions of this article will be made available by the authors upon request.

## Abbreviations

The following abbreviations are used in this manuscript:

SARS-CoV	Severe Acute Respiratory Syndrome Coronavirus 2
PBS	Phosphate-Buffered Saline
cDNA	Complementary Deoxyribonucleic Acid
